# The many mysteries of *Homo naledi*

**DOI:** 10.7554/eLife.10627

**Published:** 2015-09-10

**Authors:** Chris Stringer

**Affiliations:** Department of Earth Sciences, Natural History Museum, London, United Kingdomc.stringer@nhm.ac.uk

**Keywords:** *Homo naledi*, hominin, Dinaledi Chamber, paleoanthropology, taphonomy, other

## Abstract

More than 1500 fossils from the Rising Star cave system in South Africa have been assigned to a new human species, *Homo naledi*, which displays a unique combination of primitive and derived traits throughout the skeleton.

**Related research articles** Berger LR, Hawks J, de Ruiter DJ, Churchill SE, Schmid P, Delezene LK, Kivell TL, Garvin HM, Williams SA, DeSilva JM, Skinner MM, Musiba CM, Cameron N, Holliday TW, Harcourt-Smith W, Ackermann RR, Bastir M, Bogin B, Bolter D, Brophy J, Cofran ZD, Congdon KA, Deane AS, Dembo M, Drapeau M, Elliott MC, Feuerriegel EM, Garcia-Martinez D, Green DJ, Gurtov A, Irish JD, Kruger A, Laird MF, Marchi D, Meyer MR, Nalla S, Negash EW, Orr CM, Radovcic D, Schroeder L, Scott JE, Throckmorton Z, Tocheri MW, VanSickle C, Walker CS, Wei P, Zipfel B. 2015. *Homo naledi*, a new species of the genus *Homo* from the Dinaledi Chamber, South Africa. *eLife* **4**:e09560. doi: 10.7554/eLife.09560; Dirks PHGM, Berger LR, Roberts EM, Kramers JD, Hawks J, Randolph-Quinney PS, Elliott M, Musiba CM, Churchill SE, de Ruiter DJ, Schmid P, Backwell LR, Belyanin GA, Boshoff P, Hunter KL, Feuerriegel EM, Gurtov A, Harrison JG, Hunter R, Kruger A, Morris H, Makhubela TV, Peixotto B, Tucker S. 2015. Geological and taphonomic context for the new hominin species *Homo naledi* from the Dinaledi Chamber, South Africa. *eLife* **4**:e09561. doi: 10.7554/eLife.09561

**Image** Fossils representing at least 15 individuals of *Homo naledi* have been found

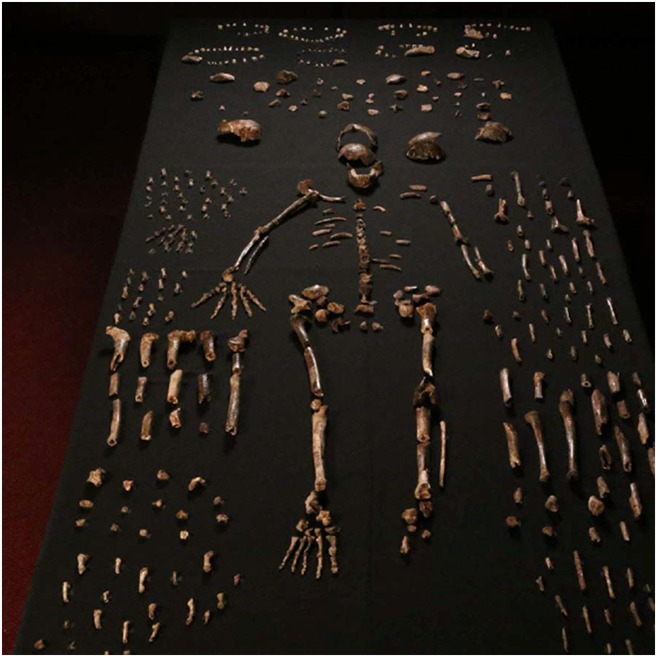

When the recovery of fossil hominin remains from the Rising Star cave system near Johannesburg in South Africa was widely publicised in 2013 and 2014, I'm sure I wasn't the only one who thought that the coverage had more hype than substance. But now, in two papers in *eLife*, we can see what the fuss was all about as Lee Berger of the University of the Witwatersrand, Paul Dirks of James Cook University and an international team of colleagues report the discovery of more than 1500 fossils that represent at least 15 individuals (Berger et al., 2015; Dirks et al., 2015). These remains have now been assigned to a new human species, which has been named *Homo naledi*. However, despite the wealth of information about the physical characteristics of *H. naledi* that this collection provides, many mysteries remain. How old are the fossils? Where does *H. naledi* fit in the scheme of human evolution? And how did the remains arrive deep within the cave system?

In the first paper, Berger et al. describe how the collection displays a unique combination of primitive and derived characteristics (Berger et al., 2015). For example, the small brain size, curved fingers and form of the shoulder, trunk and hip joint resemble the prehuman australopithecines and the early human species *Homo habilis*. Yet the wrist, hands, legs and feet look most like those of Neanderthals and modern humans. The teeth have some primitive features (such as increasing in size towards the back of the tooth row), but they are relatively small and simple, and set in lightly built jawbones (Figure 1). Overall, to my eye, the material looks most similar to the small-bodied examples of *Homo erectus* from Dmanisi in Georgia, which have been dated at ∼1.8 million years old (Lordkipanidze et al., 2013). However, the rich *H. naledi* sample includes bones that are poorly known in other early humans species such as *Homo rudolfensis,* *H. habilis* and *H. erectus*, so it is difficult at the moment to assess how similar these species were throughout the skeleton.

**Figure 1.**
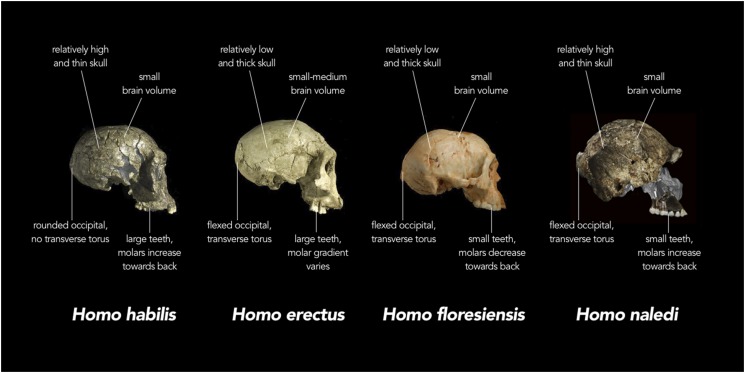
Comparison of skull features of *Homo naledi* and other early human species. Replica crania of (left to right) *Homo habilis* (KNM-ER 1813, Koobi Fora, Kenya ∼1.8 million years old), an early *Homo erectus* (D2700, Dmanisi, Georgia ∼1.8 million years old) and *Homo floresiensis* (Liang Bua 1, Indonesia ∼20,000 years old) are compared with actual fragments of cranial material of *H. naledi* that have been overlaid on a virtual reconstruction (far right; note some of the images of *H. naledi* material have been reversed). In each case, the crania are labelled with the typical features of each species. For example, while the adult brain volume of modern humans (*Homo sapiens*) is typically between 1000 and 1500 cubic centimetres (cc), *H. habilis* ranged from about 510 to >700 cc, *H. erectus* from about 550 to >1100 cc, *H. floresiensis* about 426 cc, and *H. naledi* between 466 and 560 cc. Furthermore, in modern humans, the occipital bone (at the back of the skull) is typically evenly rounded in profile, whereas in some early humans such as *H. erectus*, the upper and lower portions of the occipital are sharply angled to each other (i.e., ‘flexed’), and there is a strong ridge of bone running across the angulated region (called a transverse torus).

If *H. naledi* is more than 2 million years old, which Berger et al. suggest could be possible, the species might lie close to the very origin of the genus *Homo*. However, if the *H. naledi* fossils are less than 100,000 years old, it would mean that it survived until relatively recently, just like *Homo floresiensis* far away in Indonesia (another species which combines a small brain with small teeth; Stringer, 2014). Because *H. naledi* is currently only known from one site, it is unclear whether or not it was restricted to southern Africa. If it turns out that *H. naledi* was more widespread, its moderate body size may lead scientists to re-examine other diminutive fossils from across Africa, which have usually been attributed to a small form of *H. erectus* (Antón, 2004; Potts et al., 2004; Simpson et al., 2008).

In the second paper, Dirks, Berger and colleagues describe the setting of the fossils: the Dinaledi Chamber (Dirks et al., 2015). This cave chamber lies some 80 metres into the Rising Star system, and must have always been in constant darkness. This closely parallels the circumstances of a famous accumulation of ∼6500 human fossils in the Sima de los Huesos (‘Pit of the Bones’) in the Sierra de Atapuerca in Spain. In both cases, there is no associated evidence of human occupation. However, unlike the Dinaledi Chamber, the Sima did contain material from other large mammals. Moreover, the Atapuerca team also recovered numerous bones of the hands, feet and spine that could be articulated (or connected back together as they were in life). This led them to propose that the remains of at least 28 early Neanderthals had been intentionally thrown down into the pit, where the bodies decayed (Carbonell and Mosquera, 2006). After considering the alternative explanations, ranging from whether *H. naledi* occupied the caves to whether the bodies were left there by predators, Dirks et al. favour a similar scenario to that proposed for the Sima. However, they also recognise that the intentional disposal of the dead bodies is a surprisingly complex behaviour for a creature with a brain no bigger than that of *H. habilis* or a gorilla

Frustratingly, the rich and informative *H. naledi* material remains undated. Given that this hominin material could conceivably even date within the last 100,000 years, I am puzzled by the apparent lack of attempts to estimate its age. This could have been achieved directly via radiocarbon dating (even if only to test whether the material lies beyond the effective range of that method) or indirectly based on ancient DNA samples. For example, after ancient DNA was successfully recovered from the Sima de los Huesos fossils, it was used to date them to about 400,000 years old (Meyer et al., 2014). Moreover, tests on even small fragments of bone and tooth enamel could have narrowed down the possible age range and at least ruled out either a very ancient or very young age (Grün, 2006).

Even without date information, the mosaic nature of the *H. naledi* skeletons provides yet another indication that the genus *Homo* had complex origins. The individual mix of primitive and derived characteristics in different fossils perhaps even indicates that the genus *Homo* might be ‘polyphyletic’: in other words, some members of the genus might have originated independently in different regions of Africa. If this is the case, it would mean that the species currently placed within the genus *Homo* would need to be reassessed (Dembo et al., 2015; Hublin, 2015). While many have concentrated on East Africa as the key and perhaps sole region for the origins of the genus *Homo*, the continuing surprises emerging from further south remind us that Africa is a huge continent that even now is largely unexplored for its early human fossils.
